# Thyroid disorders induced by immune checkpoint inhibitors

**DOI:** 10.1007/s12020-024-03718-2

**Published:** 2024-02-12

**Authors:** Dimitra Karaviti, Eleni-Rafaela Kani, Eleftheria Karaviti, Eleni Gerontiti, Olympia Michalopoulou, Katerina Stefanaki, Paraskevi Kazakou, Vasiliki Vasileiou, Theodora Psaltopoulou, Stavroula A. Paschou

**Affiliations:** 1 Endocrine Unit and Diabetes Center, Department of Clinical Therapeutics, Alexandra Hospital, School of Medicine, National and Kapodistrian University of Athens, Athens, Greece; 2 Department of Endocrinology, Alexandra Hospital, Athens, Greece

**Keywords:** Immune checkpoint inhibitors, Immune-related adverse events, Thyroid dysfunction, Pathogenesis, Diagnosis, Management

## Abstract

Immune checkpoint inhibitors (ICIs) are a revolutionary class of drugs that powerfully contribute to cancer therapy by harnessing the immune system to fight malignancies. However, their successful use as anti-cancer drugs is accompanied by a wide spectrum of immune-related adverse effects (irAEs), including endocrinopathies. Among them, thyroid dysfunction stands out as one of the most common endocrinopathies induced by ICI therapy and surfaces as a prominent concern. Destructive thyroiditis is the pathophysiological basis shared by the most common patterns of thyrotoxicosis followed by hypothyroidism and isolated hypothyroidism. Diagnostic approach is guided by clinical manifestation, laboratory evaluation and imaging modalities. Treatment approaches range from the substitution of levothyroxine to the utilization of beta blockers, depending on the extent of thyroid dysfunction’s severity. While the medical community is dealing with the evolution and complexities of immunotherapy, recognizing and effectively managing ICI-induced thyroid dysfunction emerged as crucial for enhancing patient safety and achieving improved outcomes. The aim of this review is to navigate the significance of ICI-induced thyroid dysfunction unraveling the various patterns, underlying mechanisms, diagnostic approaches, and treatment strategies. It, also, highlights the impact of various factors such as cancer subtype, ICI dosage, age, and genetic susceptibility on the risk of experiencing dysfunction.

## Introduction

In recent years, immune checkpoint inhibitor (ICI) therapy has emerged as a promising approach in cancer treatment. This breakthrough approach leverages the body’s immune system to fight cancerous tumors, providing patients with valuable therapeutic alternatives and improved survival prospects [1]. Initially employed in the treatment of malignant melanoma and lung cancer, this technique involves the administration of monoclonal antibodies that target specific cell proteins such as cytotoxic T lymphocyte-associated protein 4 (CTLA-4), programmed cell death protein 1 (PD-1), and its ligand (PD-L1). Therefore, T cell activation against neoplasms is induced [2, 3]. However, alongside its benefits, the therapy comes with a range of adverse effects, with thyroid disorders being among the most prevalent endocrine complications. Thyroid dysfunction manifests with a wide clinical spectrum encompassing thyroiditis, both hypo- and thyrotoxicosis, Graves’ disease [4]. Its pathophysiological basis is considered to be destructive thyroiditis induced by a T cell-mediated acute autoimmune response [1, 5–9]. However, studies consistently highlight the role of autoantibodies against thyroglobulin (Tg), thyroid peroxidase (TPO) and thyroid stimulating hormone (TSH) receptor and even the role of cytokines in the pathogenesis of the disease [10, 11]. Therefore, laboratory tests involving TSH, free thyroxine (fT4), and antibody measurements carry immense importance not only in accurate diagnosis, but also as check-up prior to and during ICI therapy [12]. ICI-induced thyroidopathy ranges in clinical presentation from asymptomatic cases to severe manifestations and death [13]. Addressing these complications necessitates the prompt diagnosis of the thyroid disorder, along with the implementation of therapeutic strategies and drug dosages, tailored to the clinical manifestations and their severity. In essence, close monitoring and collaboration between oncologists and endocrinologists is required [13–15].

## Pathogenesis

Thyroid dysfunction is the most common endocrine adverse effect associated with ICI therapy. Most studies report two patterns of ICI-related thyroid dysfunction: thyrotoxicosis followed by hypothyroidism and isolated hypothyroidism. However, the pathophysiological basis that appears to be common is destructive thyroiditis [5–7, 9]. The underlying pathophysiology is considered to be an immune-mediated acute inflammation followed by destruction of the thyroid gland. ICI therapy induces autoimmune side effects through T cell activation and is characterized by intra-thyroidal predominance of CD8+ and CD4-CD8- T lymphocytes [1, 8]. In a case report of a nivolumab-related hypothyroidism, the anti-PD-L1 therapy suppressed the inhibitory PD-1/PD-L1 signals on follicular helper T cells (Th), leading to increased proliferation and subsequent overproduction of thyroid autoantibodies [16].

In accordance with the above, recent studies suggest that autoantibodies against thyroid peroxidase (TPOAb) and thyroglobulin (TgAb) have been found elevated at baseline in some patients who develop thyroid dysfunction after ICI immunotherapy. TPOAb and TgAb may be present at baseline prior to or may develop after ICI therapy. Maekura et al. studied the levels of TPOAb and TgAb in 53 patients treated with nivolumab for non-small cell lung cancer (NSCLC) in an attempt to predict the occurrence of hypothyroidism [17]. Among the nine patients who tested positive for TPOAb at baseline, 44% (4 out of 9) developed ICI-related hypothyroidism, compared to 2% (1 out of 44) in those who were TPO Ab negative at baseline. Among the nine patients who had positive TgAb at baseline, 56% (5 out of 9) developed ICI-related hypothyroidism, while no one out of the 44 patients who were TgAb negative at baseline experienced the same. In the Osorio et al. study, TPOAb and TgAb levels were examined not only at baseline, but also during pembrolizumab (anti-PD-1) treatment [18]. Additionally, according to the study of Muir et al. anti-thyroid antibodies basal positivity is associated with increased possibility of developing a thyroid disorder. The risk of overt thyrotoxicosis is higher when the title of TPOAb or/and TgAb is remarkably elevated or when there is a newfound thyroid antibody positivity [19]. The correlation between basal positivity of TPOAb and TgAb and high risk of thyroid dysfunction after the initiation of ICIs therapy is also supported by the study of Zhou et al. [20]. Reportedly, the development of positive thyroid autoantibodies after initiation of ICI therapy is associated with higher risk of ICI-induced thyroid dysfunction.

In similar studies, stimulatory autoantibodies against TSH receptor (TRAb) or thyroid-stimulating immunoglobulin (TSI) predominantly found negative in the majority of cases. Case reports indicate that ICI therapy-induced thyroid dysfunction may impact and abolish the thyroid stimulating effect of TSI. Notably, destructive thyroiditis may coexist with Graves’ disease in a minority of cases, as suggested by TRAb positivity [10, 11].

Individual genetic susceptibility to thyroid dysfunction also plays an important role. More specifically, thyroid dysfunction has been shown to be associated with overexpression of Human Leukocyte Antigen DR-isotype (HLA-DR). Treatment with ICI therapy can change HLA-DR expression, increasing T cell activation and leading to thyroid autoimmune disease. Delivanis et al. conducted a study examining healthy volunteers, patients with autoimmune thyroiditis, and individuals with pembrolizumab-induced thyroiditis [21]. The study revealed an elevated count of CD56 + CD16+ Natural Killer (NK) cells and increased surface HLA DR expression on inflammatory intermediate CD14 + /CD16+ monocytes in patients with pembrolizumab-related thyroiditis. Comparing the PD-1 levels on peripheral T-cells among the three groups, they were undetectable on the surface of T-cells in those with pembrolizumab-induced thyroiditis, while they were comparable between healthy volunteers and patients with autoimmune thyroiditis. Thus, macrophage activation through up-regulation of HLA-DR may be a possible mechanism of pembrolizumab-induced thyroiditis. In addition to T and B lymphocytes, various cytokines play an essential role in the development of thyroid disorders. First and foremost, elevated interleukin (IL)-2 levels facilitate the binding between HLA-II and thyroid cell autoantigen, leading to stimulation of CD8+ cytotoxic T lymphocytes (CTL) and subsequent thyroid cell death. According to recent studies PD-L1 therapy increases CD4 + Th1 and therefore the expression of interferon gamma (IFN-γ) and IL-2, which leads to thyroid cell death. Kurimoto et al. measured the changes of various cytokines before and after ICIs treatment and identified that an increase in IL-2 and a decrease in granulocyte colony-stimulating factor (G-CSF) appeared to be associated with thyroid immune-related adverse events (irAEs) [22]. Regulatory T cells (Tregs) enhance the response to anti-PD-L1 therapy by releasing cytokine IL-10. Conversely, Treg inhibition through ICI therapy is implicated in the development of autoimmune thyroid diseases [23] (Table 1).

**Table 1:** Thyroid dysfunction linked with particular ICI-therapy for specific cancer subtypes

Study	Publication year	Type of study	Objective	Cancer type	Type of ICI-therapy	Thyroid disorder induced	Outcomes/Findings
D.L. Morganstein et al. [5]	2016	Retrospective observational study	Evaluate thyroid dysfunction and its progression in ICI-treated melanoma patients.	Melanoma	Anti-PD-L1, anti-PD-1, anti-CTLA-4 or combination of ipilimumab- nivolumab	Hypothyroidism, thyrotoxicosis	TD can affect as many as half of the patient. F > M
Lee H. et al. [6]	2017	Cohort study	Comparison of thyroid disorders between patients receiving different treatment regimens	Solid and hematologic malignancies	Anti-PD-1 or anti-PD-1 and anti- CTLA-4 combination	Hypothyroidism, thyrotoxicosis	Most common ir-TD: thyroiditis. Εarlier onset of thyrotoxicosis-, hypothyroidism later
Jeroen de Filette et al. [9]	2016	Observational, cohort study	Examine the occurrence and attributes of thyroid dysfunction related to pembrolizumab	Melanoma	Pembrolizumab (anti-PD-1)	Thyrotoxicosis, hypothyroidism	Thyrotoxicosis is associated with diffuse increased 18 FDG uptake
DA Delivanis et al. [21]	2017	Single- center, retrospective cohort study	Investigate the incidence and the possible causes of thyroid irAEs triggered by anti-PD-1 treatment	Metastatic melanoma, NSCLC	Pembrolizumab (anti-PD-1)	Destructive thyroiditis and overt hypothyroidism	The mechanism of thyroid damage seems unrelated to thyroid autoantibodies and might involve pathways influenced by T cells, NK cells, and/or monocytes.
SA Paschou et al. [33]	2022	Cohort study	Investigate the relation of endocrine irAE with PFS and OS	Various cancer types: bladder, renal, lung, ovarian cancer	Anti-PD1, anti-PD-L1	Non-specific	Endocrinopathies induced by ICIs could potentially serve as indicators of a favorable response to immunotherapy.
Osorio JC et al. [18]	2016	Observational cohort study	Examine TD in NSCLC patients treated with pembrolizumab, association with antibodies and impact on survival	NSCLC	Anti-PD-1	Transient thyrotoxicosis, hypothyroidism	Thyroid dysfunction occurs early in the pembrolizumab course and may be associated with improved outcomes.
F. Guaraldi et al. [7]	2017	Long-term prospective single-arm study	Evaluation of the occurrence and significance of ICI-induced thyroid disorders in actual clinical practice	Melanoma	Anti-CTLA-4 (ipilimumab), Anti-PD1 (nivolumab, pembrolizumab)	Hypothyroidism, thyrotoxicosis	ICIs are associated with high occurrences of severe autoimmune TD. Autoimmune conditions and BRAF mutation are linked to improved clinical responses after anti-CTLA-4 followed by anti-PD1
Olsson-Brown A et al. [25]	2020	Retrospective observational study	Characterization of the clinical patterns of thyroid dysfunction to cancer patients treated with ICIs	Metastatic malignant melanoma	Anti-PD1 mono/combination with anti-CTLA-4	hypothyroidism, thyrotoxicosis followed by hypothyroidism	No correlation between clinical pattern of dysfunction and thyroid antibodies, gender association: F > M
Difei Lu et al. [26]	2022	Retrospective analysis	Analysis of ICI-TD prevalence and demographics, along with identification of poor clinical outcome risk factors, using FAERS data.	broad subtype of malignancies	Anti-PD1, anti-PD-L1, anti-CTLA-4, combination	Hypothyroidism, thyrotoxicosis	ICI-TDs are presented as either hypothyroidism or thyrotoxicosis, particularly in individuals undergoing combination therapy.
Husebye ES et al. [1]	2022	Guideline	Provision of evidence-based recommendations for treatment and follow-up for ICI-induced endocrinopathies addressing the gaps in existing guidelines	Broad subtype of tumors as ICIs have been approved in 18 cancer types	Anti-PD-1/PD-L1, anti-CTLA4	Hypothyroidism, thyrotoxicosis, Graves’ disease and thyroid eye disease	Provision of practical guidance and recommendations on the management of patients with ICI-related endocrine conditions.
Toshiya Maekura et al. [17]	2017	Research study	Evaluation of thyroid dysfunction and identification of the predictive factors of nivolumab-induced hypothyroidism	NSCLC	Nivolumab (anti-PD1)	Hypothyroidism	Assessing TPO and TgAb at the outset could be predictive for the development of hypothyroidism in NSCLC patients.
Yang S et al. [38]	2020	Observational cohort study	Examination of autoimmune disease risk in lung cancer patients under ICI-treatment versus chemotherapy, with a specific focus on hypothyroidism	Lung cancer	Nivolumab, Pembrolizumab (PD-1-inhibitors)	Hypothyroidism	Patients under ICI-therapy were 1.97 more likely to experience autoimmune diseases within the initial 6 months compared to chemotherapy.
Anupam Kotwal et al. [8]	2020	Single-center prospective cohort study	Investigation of immune mechanisms and genetic factors associated with TD caused by ICIs, focusing on PD-1 inhibitors	Diverse malignancies (most common: melanoma)	Anti-PD-1 Anti-PD-L1	Thyrotoxicosis, overt hypothyroidism following thyrotoxicosis, primary hypothyroidism	Thyroiditis induced by ICIs is associated with a notable presence of CD8+ and CD4 − CD8 − T lymphocytes to the thyroid tissue. HLA haplotypes might play a role.
Kurimoto et al. [22]	2020	Prospective clinical observational research	Identification of predictive and sensitive biomarkers in thyroid irAE.	Melanoma, RCC, NSCLC, UTUC, gastric cancer	Anti-PD1 (pembrolizumab, nivolumab), anti-CTLA-4 (ipilimumab) or combination	Thyrotoxicosis, hypothyroidism	Potential biomarkers: serum Tg, TgAb, TPOAb, IL-1β, IL-2, GM-CSF, IL-8, MCP-1

*TD* thyroid disorder, *irAE* immune-related adverse events, *irTD* immune related thyroid disorders, *NK cells* Natural killer cells, *PFS* progression-free survival, *OS* overall survival, *Anti-PD1* anti-programmed death r-1, *Anti-PD-L1* anti-programmed death ligand-1, *Anti-CTLA-4* anti-cytotoxic T-lymphocyte-associated protein 4, *ICI-TD* immune checkpoint inhibitor-related thyroid dysfunction, *TD* thyroid disorders, *FAERS* FDA Adverse Event Reporting System, *UTUC* urothelial carcinoma, *RCC* renal cell carcinoma, *NSCLC* non-small cell lung cancer, *F* *>* *M* females> Males, *Tg* thyroglobulin, *TgAb* thyroglobulin antibodies, *TPOAb* thyroperoxidase antibodies

## Clinical presentation

Clinical diagnosis of thyroid toxicity is challenging, as patients may not exhibit any noticeable symptom or sign or present with non-specific symptoms [24]. However, due to the accessibility of thyroid function screening, thyroid disorders are frequently identified at an early stage, even when patients do not display typical symptoms. The spectrum of thyroid disorders induced by ICIs includes thyroiditis, hypothyroidism, Grave’s disease and thyrotoxicosis [6]. Most cases involve thyroiditis processing to hypothyroidism [4]. Although hypothyroidism is typically permanent in most cases, it is currently impossible to determine the ratio of transient hypothyroidism compared to permanent cases [25, 26].

For the majority of symptomatic patients, the first manifestation is thyrotoxicosis [6]. Thyrotoxicosis usually presents with weight loss, palpitation, tremors, anxiety, fatigue and sweating [27]. In addition, increased perspiration, heat intolerance, hyperdefecation and generally increased metabolic activity are clinical manifestations that should raise suspicion of thyrotoxicosis [28, 29]. Physical examination sometimes reveals increased heart race and warm skin [13]. Atrial fibrillation may be seen, especially in older patients [30]. Initial presentations of overt or subclinical thyrotoxicosis typically resolve to euthyroidism or hypothyroidism within several weeks to months.

Primary hypothyroidism is a thyroid adverse effect that is more commonly noticed in patients treated with PD-1 inhibitors [31]. Hypothyroidism can be detected during routine lab monitoring in asymptomatic patients or clinically presents with the typical symptoms such as weight gain, depression, profound fatigue, alopecia, cold intolerance, constipation, dry skin, bradycardia, periorbital edema and tongue swelling [4, 29, 30]. While most cases are mild to moderate, untreated severe hypothyroidism can lead to myxedema coma, decreased mental status and often hypothermia [30].

Graves’ disease induced by ICIs is primarily associated with CTLA‐4 gene polymorphisms [32]. While it is extremely rare, cases have been reported. It appears usually at the beginning of the treatment. The presence of thyroid eye disease increases the possibility of Graves’ disease, while signs such as orbitopathy or a large goiter enhance its diagnosis [27, 33].

## Epidemiology

Thyroid disorders may occur even after the first single therapeutic dose of ICI therapy [6, 13]. The median time of the onset is 6–10 weeks after the initiation, but it may happen as early as 7 days post therapy initiation and as late as 3 years [6, 13, 21, 33–35]. Thyroid dysfunction is mainly associated with anti-PD-1 monotherapy and its combination with PD-L1 or/and anti-CTLA-4 therapy rather than with anti-CTLA-4 or anti-PD-L1 monotherapy [19, 27, 36]. CTLA-4 inhibitors are mostly correlated with the possibility of developing hypothyroidism, while PD-1/PD-L1 inhibitors can lead to thyrotoxicosis and hypothyroidism [19]. More specifically, thyrotoxicosis induced by anti-CTLA-4 affects 0.2–1.7% of the patients, while thyrotoxicosis induced by anti-PD-1 affects o.6-3.7% of the patients. ICIs combination is responsible for 8–11% of the cases of thyrotoxicosis. Hypothyroidism is induced by CTLA-4 inhibitors in 2.5–5.2% of the cases, by PD-1/PD-L1 inhibitors in 3.9-8.5% and by the combination of anti-CTLA-4 and anti-PD-1 in 10.2–16.4% of the patients [37]. Various factors, including cancer subtype, the ICI dosage, and age, influence the risk of thyroid ICI side effects, with current conflicting results regarding age and sex hormones [38, 39]. ICIs induce thyroid dysfunction more frequently in women than in men [28].

## Thyroid disorders induced by ICIs and cancer prognosis

Several studies have shown that cancer patients undergoing ICI treatment who develop immune-related adverse effects (irAEs), particularly thyroid dysfunction, often exhibit improved prognosis. Combining the results of the retrospective studies conducted by Prather et al. and Trudu et al., it is suggested that lung cancer patients experiencing irAEs had longer progression-free survival (PFS) and improved overall survival compared to those without these side effects. This implies that irAEs may serve as potential indicators of enhanced treatment efficacy [40, 41]. Similarly, Zheng et al. reported that 47% of hepatocellular carcinoma (HCC) patients treated with anti-PD-1 therapy developed thyroid dysfunction [42]. The survival rates showed no significant difference between the group with normal thyroid function and the one with abnormal thyroid function [42]. Han-Sang Baek also revealed that individuals experiencing with irAEs, in particular hypothyroidism, demonstrated a more favorable prognosis compared to those without irAEs. This association remained irrespective of factors such as age, sex, type of ICI used, and cancer type [43]. Studies by Kotwal et al. and Lima Ferreira et al. also observed improved survival in patients with thyroid dysfunction across different cancer types and ICI therapies [44, 45]. However, these studies did not distinguish between different types of thyroid dysfunction. In contrast to these findings, a case study highlighted a lung cancer patient who developed ICI-related thyroid dysfunction, leading to tumor progression and preventing surgical intervention. This suggests that thyroid dysfunction does not uniformly indicate a better response to ICI treatments [43, 46]. This becomes more intricate as studies propose that thyrotoxicosis could potentially exacerbate cancer prognosis [47]. Von Itzstein et al. noted poorer outcomes in patients with pre-existing thyroid dysfunction but also observed that initiating levothyroxine after beginning ICI treatment improved overall survival [48]. This indicates that pre-existing thyroid problems might negatively impact the effectiveness of ICI therapy and should be managed adequately before starting ICI treatment.

## Laboratory and imaging evaluation

The evaluation of TSH and fT4 is recommended before the initiation of ICI therapy, in order to rule out the possibility of any preexisting thyroid disorder [12]. Thyroid function screening, involving TSH and fT4 measurements, should be conducted every 4–6 weeks, or more frequently, if necessary, for all the patients undergoing ICIs treatment [4, 12, 35]. Monitoring of the patients on ICIs immunotherapy should occur 4–6 weeks after the completion of the last cycle [49]. Some clinicians suggest the measure of TSH and fT4 before every circle of treatment [14, 35].

ICI-induced hypothyroidism is characterized by decreased levels of free thyroxine (fT4) [28]. The assessment of TSH is a more sensitive test [35]. Elevated TSH with low or low-to-normal fT4 set the diagnosis of primary hypothyroidism, while low or inappropriately low-to-mid-normal TSH levels with low fT4 indicate secondary/central hypothyroidism attributed to pituitary disorder, such as hypophysitis [12, 30, 50, 51]. In this case, measuring cortisol is indicated to assess for adrenal insufficiency [30]. Primary hypothyroidism may be subclinical. Subclinical hypothyroidism is diagnosed when TSH is elevated but below 10 mIU/mL accompanied by a normal free T4 level [4]. For cases of hypothyroidism, including TPO-antibody testing is prudent as it indicates an autoimmune origin [34].

Overt thyrotoxicosis is characterized by suppressed TSH serum levels and elevated fT4 and/or total triiodothyronine (TT3), while subclinical thyrotoxicosis is defined as suppressed TSH with normal fT4 and TT3 serum levels [9]. Due to the potential process to subclinical hypothyroidism, a repeat thyroid function evaluation test should be performed 6 weeks after the initial diagnosis [4]. Guidelines recommend TSH receptor-antibody testing in case of thyrotoxicosis [34].

Suspecting Graves’ disease is raised when thyroid hormones (T4 and T3) levels are significantly high, initial symptoms are prominent, thyrotoxic manifestations persist for over 6 weeks and other characteristic signs, including orbitopathy or a large goiter, are present. For those patients, the diagnosis is established by the positivity TSI and/or thyrotropin receptor antibodies [12]. In contrast to screening of thyroid function, the screening for TPO-antibodies before the initiation of ICIs is not recommended [34]. Additionally, since some cases of Graves’ disease have been reported with normal levels of TSH receptor antibodies, ultrasonography and scintigraphy/gamma scan are indicated [12, 24]. Doppler ultrasound in Graves’ disease typically reveals increased blood flow, presenting as high vascularity in the thyroid gland [27]. Thyroid uptake tests are recommended for patients with high likelihood of developing Grave’s disease and who have not been exposed to intravenous CT contrast for at least 1 month, as this exposure may reduce thyroid iodine uptake [6, 27]. Radioactive iodine uptake is a valuable tool in differentiating Graves’ disease from other causes of thyrotoxicosis such as destructive thyroiditis [51]. It involves administering a diagnostic dose of iodine-123 orally, followed by measuring its absorption by the thyroid gland either 6 or 24 h later. Graves’ disease is characterized by high, diffuse, homogenous iodine uptake and elevated titers of TRAbs, while destructive thyroiditis is associated with low iodine uptake and the presence, though not elevated, titers of TRAb [15, 24, 27, 50]. Characteristic ultrasonic indicators of ICI-induced destructive thyroiditis include widespread thyroid gland enlargement, decreased internal blood flow, and reduced internal echogenicity (Table 2).

**Table 2:** Clinical presentation, laboratory and imaging evaluation of thyroid disorders

Thyroid disorder	Clinical symptoms	Physical examination	*TSH levels	**fT4, ***T3	Anti-thyroid antibodies	Imaging study
Hypothyroidism	Fatigue, weight gain, cold intolerance, constipation, depression, dry skin, facial puffiness, periorbital edema, tongue swelling	Bradycardia, hypothermia	Elevated TSH	Normal, low f4, fT3	Anti-TPO (thyroid peroxidase) testing is recommended as it suggests an autoimmune origin	(not necessary)
Thyrotoxicosis	Weight loss, anxiety, fatigue, increased frequency of bowel movements	Tremors, warm and smooth skin, palpitations, lid lag	Decreased or suppressed TSH	Elevated fT4 and/or T3 [levels of fT4 are more elevated compared to T3 levels]	Possibility of positive (+) TPO-TG Ab TSH receptor antibodies testing is recommended	Radioiodine uptake testing: decreased uptake
Graves´ disease	Ophthalmopathy/ orbitopathy (conjunctival redness, eye pain)	Thyroid bruit, large goiter	Low TSH	significantly elevated	**** [Positive (+) TSH receptor antibodies (TRAb)/TSI], possibility of positive (+) TPO-TG Ab	Doppler ultrasound reveals increased blood flow. Radioactive iodine uptake: increasing and homogeneously diffuse

*TSH* thyroid-stimulating hormone, *fT4* free thyroxine, *T3* Triiodothyronine

*TSH normal levels: 0.4–4 mUI/L

**fT4normal levels: 12–22 pmol/L

***T3 normal levels: 3.1–6.8 pmol/L

**** Cases of Graves’ disease without elevated levels of TSH have been described

## Treatment

Multiple organizations have proposed various treatment approaches for thyroid immune-related adverse events (irAEs) caused by ICIs, such as American Society of Clinical Oncology (ASCO), National Comprehensive Cancer Network (NCCN), Society for Immunotherapy of Cancer (SITC) and European Society for Medical Oncology (ESMO) (Table 3). These guidelines demonstrate a high level of agreement [13, 52]. The severity of each side effect is classified into five grades based on the Common Terminology Criteria for Adverse Effects (CTCAE) established by the National Cancer Institute (NCI) of the National Institutes of Health (NIH).

**Table 3:** Guidelines for ICI-induced thyroid disorders’ treatment

	ESE 2022 [1]	ESMO 2021 [13]	ASCO 2022[15]	SCIT 2017 [54]	British Society for Endocrinology 2018 [56]	IDSC 2018 [52]	French Endocrine Society 2019 [56]	NCCN 2019 [14]	Japanese Endocrine Society 2019 [55]
Hypothyroidism	Search for TPO-Abs (autoimmunity) ; Give levothyroxine until TSH levels are normal	Grade 1*: Continue ICI therapy; monitor TSH every 4–6 weeks. Grade 2: ** Cease ICIs until adverse effects subside; start ~1.1 μg/kg/day levothyroxine. (For elderly or cardiovascular comorbidities: gradually increase 25–50 μg initial dose); monitor TSH every 6 weeks and FT4 to confirm dose sufficiency. Grade 3: *** Manage similarly as in Grade 2**; advise endocrinologist. Grade 4: **** Manage similarly as in Grade 2**; hospitalization may be necessary for i.v. treatment in the case of myxedema.	Grade 1*: Continue ICI therapy; monitor TSH every 4–6 weeks. Grade 2: ** Hold ICIs until adverse effects subside; start levothyroxine if symptomatic or if TSH >10 mIU/L persists, monitor TSH every 6 weeks, adjusting the dose until TSH returns to normal. check FT4 to confirm dose sufficiency. Once successfully treated, resume ICI therapy. Monitor THs every 6 weeks during therapy and yearly after therapy. Grade 3: *** Manage similarly as in Grade 2**; advise endocrinologist. Grade 4: **** Manage similarly as in Grade 2**; hospitalization may be necessary for i.v. treatment in case of myxedema.	Grade 3*** and 4 ****: Cease ICIs; hospitalization may be necessary for grade 4**** patients. Grade 2: ** Postpone ICIs for symptomatic patients, until symptoms resolve. Give 1.6 mcg/kg levothyroxine (except from elderly and patients with cardiovascular comorbidities who should start with a lower dose of 25–50 μg) and monitor TSH and fT4 every 6-8 weeks until normal TSH levels achieved; If TSH remains high, increase levothyroxine dose by 12.5 mcg to 25 mcg; maintain the dose; reassure after 12 months or earlier if the patient’s condition alters	If myxedema is suspected, advise an endocrinologist; Exclude first adrenal crisis and then investigate T4; If in doubt, treat for cortisol deficiency	If ICI therapy includes CTLA4 blockade and TSH is normal or low, exclude firstly secondary adrenal insufficiency, after euthyroid sick syndrome; otherwise levothyroxine is indicated	ICI therapy is not contraindicated, but can be carried over for later; If TSH > 10 miU/L, administer levothyroxine at an initial dose 1–1.6 µg/kg/day; Adjust the dose according to patient’s age, comorbidities and survival prognosis; If TSH 5–10 miU/L and anti-TPO antibodies or symptoms are present, consider giving levothyroxine; In case of hypothyroidism following a destructive thyroiditis, thyroid hormones may be needed lifelong	Grade 1*: Continue ICI therapy; monitor TSH every 4–6 weeks; Grade 2: ** Cease ICIs until adverse effects subside; Start ~1.6 mcg/kg/day levothyroxine (For elderly and patients with cardiovascular comorbidities: reduce dose by ~10%); Advise endocrinologist; Grade 3: *** Hold ICIs in any case and manage similarly as mentioned right above; Grade 4: **** Manage similarly as mentioned right above;	Hold ICI therapy; Administer an initial dose of 25–50 μg levothyroxine per day except from elderly or patients with cardiac problems who should start with a dose of 12,5 μg; Continue treatment adjusting the dose in regard to the serum TSH levels
Thyrotoxicosis	Search for TRabs - exclude Grave’s; administer non-selective beta-blocker to alleviate symptoms;Give corticosteroids in high dosages; if symptoms persist ( > 6 weeks), search again for TRAb, test fT3, conduct ultrasound or scintigraphy to exclude Graves’ ; if Grave’s give antithyroid drugs	Grade 1*: Continue ICI therapy. Monitor TSH, FT4, T3 every 2–3 weeks. Recognize destructive thyroiditis or persistent thyrotoxicosis Grade 2**: Hold ICIs until adverse effects subside; administer a beta-blocker and methimazole if thyrotoxicosis persists; measure TSH, FT4, T3 every 4–6 weeks; advise endocrinologist. Grade 3 ***: Treat similarly as mentioned above. Consider corticosteroids. Grade 4 ****: Hospitalization may be needed for i.v. treatment. Start 1 to 2 mg/kg/d prednisone while titrating for 1 to 2 weeks. Consider saturated solution of potassium iodide or PTU/methimazole. Cease ICI at grade 3 and grade 4****.	Grade 1*: Continue ICI therapy, monitor TSH, FT4, T3 every 2–3 weeks, recognize destructive thyroiditis or persistent thyrotoxicosis Grade 2**: Hold ICIs until adverse effects subside; administer beta-blocker and methimazole if needed, measure TSH, FT4, T3 every 3–4 weeks, advise endocrinologist; hydrate and supportive care; Investigate for Grave’s if thyrotoxicosis persists > 6 weeks; Grade 3 ***: Treat as in Grade 2**, consider corticosteroids. Grade 4 ****: Hospitalization may be necessary, start 1 to 2 mg/kg/d prednisone while titrating for 1 to 2 weeks; Saturated solution of potassium iodide or PTU/methimazole can also be used	If thyrotoxicosis is suspected, exclude Grave’s disease firstly (requires thionamides); Management without many medical interventions effectively; give non-selective b blocker (with alpha blocker action preferably) in symptomatic patient; TFT test every 2 weeks; in case evolvement into hypothyroidism, levothyroxine; Cease ICIs at grade 3*** and grade 4**** patient. At grade2**, hold ICIs until adverse effects subside; Advise endocrinologist;	If thyrotoxicosis is suspected or is present, provide supportive care in an ICU/ a HDU and endocrinologist’s supervision	Differentiate thyroiditis from Grave’s; Monitor closely TSH, T4, T3 in case of hypothyroidism appearance or persistence of thyrotoxicosis; if thyrotoxicosis persists, check for TSI or scan for iodine uptake	Treatment based on appointment between the oncologist and endocrinologist; ICI therapy is not contraindicated, but can be carried over for later; if asymptomatic, no intervention, just close monitoring; if symptoms are present, administer b blocker: consider corticosteroids for severe cases. Give levothyroxine while continuing the ICI therapy after thyrotoxicosis, reduce gradually the dose as long as the therapy comes to an end; simultaneously close monitor TSH levels	Give non-selective b blockers to alleviate symptoms, glucocorticoids in high dosages and PTU. Monitor TSH and FT4 levels 4–6 weeks after the crisis; if abnormal, administer I-123 or scan to identify the cause of thyrotoxicosis and rule out Graves’ disease; if thyrotoxicosis turns into hypothyroidism, give 1.6 mcg/kg/day levothyroxine orally until TSH reaches normal levels	Hold ICI therapy; give b blockers as symptoms relievers e.g., propranolol 30 mg per day
Thyroid-like eye disease (TED)	n/a recommendation for holding or continuing ICIs; Give systemic corticosteroids in high dosages;	Hold ICIs only in severe forms of TED; give systemic corticosteroids in high dosages;	n/a	n/a	n/a	n/a	Hold ICI therapy in case of severe orbitopathy; the decision to continue the therapy should be taken personalized for each case	n/a	n/a

Severity grades are based on the Common Terminology Criteria for Adverse Effects (CTCAE) established by the National Cancer Institute (NCI) of the National Institutes of Health (NIH):

* TSH < 4-10mIU/l, FT4: normal and without symptoms

** TSH >10mIU/l or TSH 4–10 mIU/l with low FT4 with or without symptoms

*** TSH>10mIU/l or TSH 4–10 mIU/l with low FT4 and severe symptoms

**** TSH > 10mIU/l with very severe symptoms (life threatening)

*n/a* non-available, *ICU* intensive care units, *HDU* high-dependency care units, *PTU* propylthiouracil, *ICI* immune checkpoint inhibitor, *FT4* free thyroxine, *TSH* thyroid-stimulating hormone, *T3* triiodothyronine, *TSI* thyroid-stimulating immunoglobulin, *TRab* TSH Receptor antibody, *fT3* free triiodothyronine

In cases of hypothyroidism, asymptomatic patients with mild thyroidopathy (TSH 4–10 mIU/l), normal FT4 (grade 1) can continue ICI therapy, with regular TSH monitoring every 4–6 weeks. Ιn patients having moderate thyroidopathy (TSH over 10 mIU/l or TSH 4–10 mIU/l) with low FT4, with or without symptoms, such as constipation, cold intolerance, fatigue, disturbances in the menstrual cycle, arthralgias, myopathy, pale and/or dry skin, thin brittle hair or fingernails, depression signs, weight gain, weakness, etc (grade 2), ICI therapy should be ceased for a while, until the irAEs subside [13]. These patients should start with a replacement dose of approximately 1,3 μg/kg/day levothyroxine. Special attention should be paid to the elderly patients or those with cardiovascular compromise, beginning with a lower daily dose of 25–50 μg and gradually increasing it or simply reducing the default dose by approximately 10%, in order to avoid the risk of thyrotoxicosis [13, 14]. TSH should be assessed every 6 weeks to determine the appropriate dose, whereas FT4 can be evaluated to certify the adequacy of the default dose [13, 53]. Patients with severe thyroidopathy and symptoms like puffiness of face, low temperature, low heart rate, slow speech (grade 3) are managed similarly to patients in grade 2 with the only difference that expert opinion from an endocrinologist is necessary, while at previous grades just recommended. Patients with life-threatening thyroidopathy, exhibiting severe symptoms that could lead to death (grade 4), are treated as mentioned above [13]. However, physicians remain particularly vigilant for signs of myxedema, such as progressive weakness, stupor, hypothermia, hypoventilation, hypoglycemia and hyponatremia. In such cases, hospitalization for intravenous treatment is necessary. Levothyroxine treatment is recommended until the TSH levels are restored to normal. Adrenal insufficiency should be ruled out if suspected [14, 51].

If the physician suspects an underlying thyrotoxicosis, patients should undergo thorough monitoring to exclude the possibility of destructive thyroiditis, which requires a distinct management approach. In such instances, due to their potential to turn to hypothyroidism, it is prudent to avoid the use of I-131 [13]. For cases of thyrotoxicosis, individuals with mild thyroidopathy (TSH less than 0.4 mIU/l), whether symptomatic or asymptomatic (grade 1), can proceed with ICIs therapy, with regular monitoring of TSH, FT4, T3 every 2–3 weeks [13, 53]. Patients with moderate thyroidopathy, low TSH and moderate symptoms, such as heat intolerance, attention deficit disorder, gastrointestinal (GI) hypermotility, insomnia, hair loss, tremor, anxiety etc., (grade 2) should temporarily discontinue ICIs until the resolution of adverse effects and simultaneously initiate a beta blocker therapy [13, 53]. Methimazole or propylthiouracil (PTU) may be administered if thyrotoxicosis persists for over 6 weeks in these patients [13, 53]. In case of persistent thyrotoxicosis, it is also prudent to investigate Graves’ disease (TSH receptor autoantibodies-TRAb). TSH, FT4, T3 should be measured at these patients every 3–4 weeks and the expert’s care (endocrinologist) should be provided [53]. The management of patients with severe thyroidopathy and severe symptoms like tachycardia and palpitations (grade 3) follows the same approach as for the previous stage. The first line treatment remains beta blocker and PTU/methimazole [13]. Corticosteroids are also acceptable at this stage, despite the lack of strong evidence for their efficacy [53]. Patients with life-threatening thyroidopathy and symptoms severe enough to potentially lead to death (grade 4) are treated similarly, with the distinction that hospitalization may be warranted, when suspecting an upcoming thyroid storm [13]. Furthermore, a common and widely accepted approach at this point involves the administration of 1–2 mg/kg/d or equivalent prednisone during a brief titrating period of 1–2 weeks. Saturated solution of potassium iodide or PTU/methimazole can also be used [53].

In case of thyroid storm, it is recommended to administer a non-selective beta blocker, typically propranolol, at doses of 40 to 80 mg every four to six hours, high dose glucocorticoids, to prevent the peripheral conversion of T4 to T3, and a starting dose of 500 to 1000 mg of propylthiouracil (PTU) continuing with a subsequent dose of 250 mg every four hours [13, 14]. Both PTU and methimazole block the production of thyroid hormones. Nonetheless, PTU is preferred over methimazole in this case, because of its additional effect of blocking peripheral conversion of T4 to T3. Immunotherapy can be continued only if the patient is asymptomatic, while TSH and FT4 levels should be monitored 4–6 weeks after the crisis [14]. If they normalize, the management can be considered complete. If not, it is recommended to search again for TSH receptor autoantibodies-TRAb, even test fT3, administer I-123 or conduct a scan to identify the cause of persistent (>6 weeks) thyrotoxicosis exclude Graves’ disease [13, 14, 52, 54]. In case of Grave’s, antithyroid drugs (methimazole or PTU in the first semester of pregnancy) and beta blocker should be administered—avoid the use of I-131 as it increases the risk of hypothyroidism. If thyrotoxicosis turns into hypothyroidism, levothyroxine should be given at a dose of 1.6 mcg/kg/day orally until TSH reaches normal levels [12, 14, 54]. Another approach of managing a thyrotoxic crisis involves minimal medical interventions, with beta blockers administered only in presence of symptoms and more frequent reassessments of TSH, FT4, i.e., typically every 2–3 weeks [54–57].

**Fig. 1:**
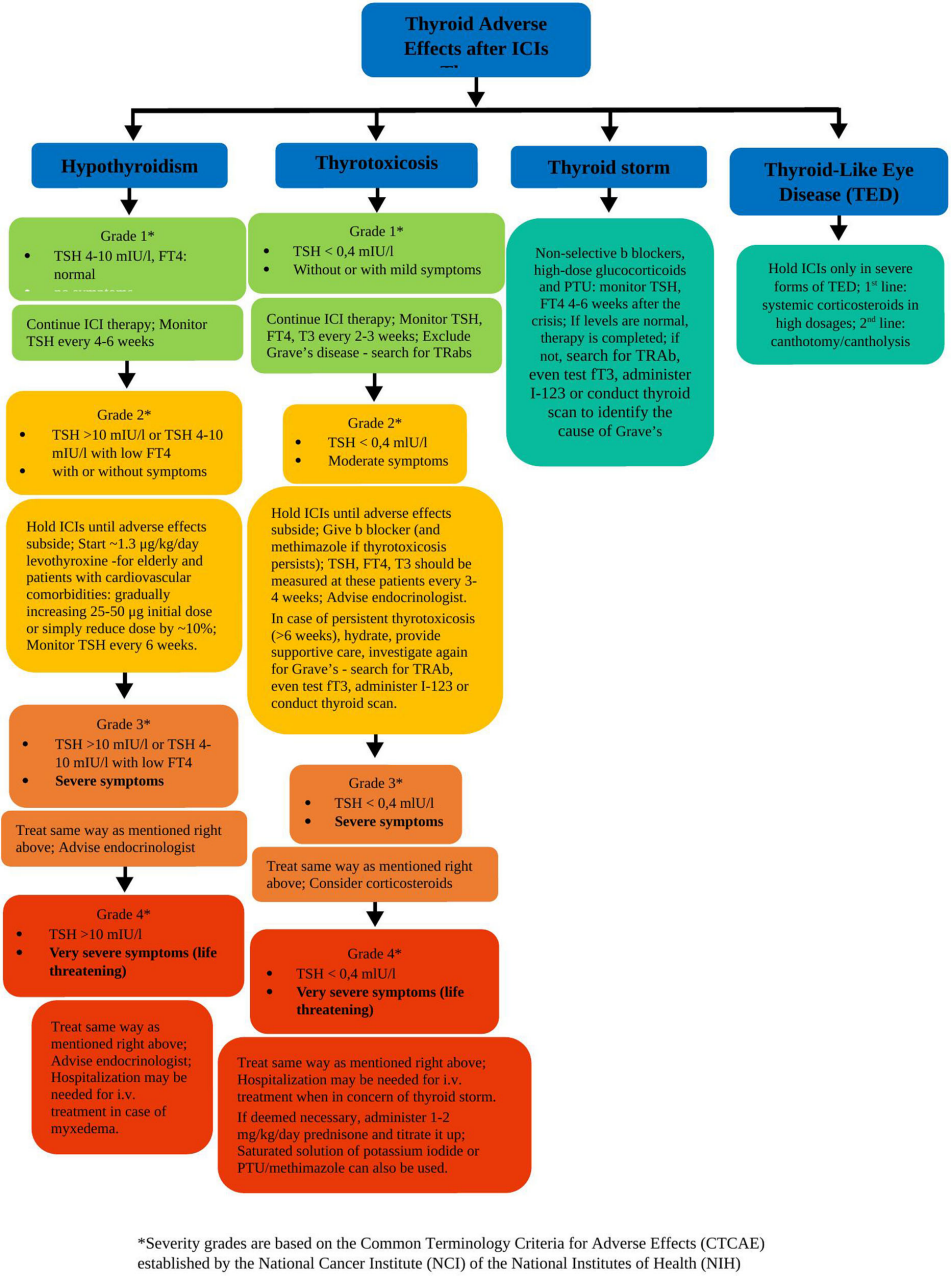
Classification of thyroid adverse effects after ICIs therapy

In case of thyroid-like eye disease (TED), high dose systemic corticosteroids are strongly recommended for their potent anti-inflammatory effect. In instances where the orbital thyroidopathy persists, canthotomy/cantholysis may be considered. As for the ICIs treatment, it should only be paused in cases of severe TED [58] (Fig. 1 and Table 4).

**Table 4:** Recommendations for managing ICI-induced thyroid disorders

Side effect	Severity	Management recommendations
Hypothyroidism	• TSH 4–10 mIU/l, FT4: normal	Continue ICI therapy; Monitor TSH every 4–6 weeks
	• without symptoms	
	• TSH > 10 mIU/l or TSH 4–10 mIU/l, low FT4	Stop ICIs if symptoms are present until they subside; Start levothyroxine;
		Measure TSH every 6 weeks for dose adjustment; Monitor fT4 in the meantime (every 2 weeks) to ensure that the management is appropriate
	• with or without symptoms	
	• TSH > 10 mIU/l or TSH 4–10 mIU/l, low FT4	Manage similarly as right above; Advise endocrinologist
	• severe symptoms	
	• TSH > 10 mIU/l	Manage similarly as right above; Hospitalization may be needed for i.v. treatment if myxedema appears
	• very severe symptoms (life threatening)	
Thyrotoxicosis	• TSH < 0.4 mIU/l	Continue ICI therapy; Monitor TSH, fT4, T3 every 2–3 weeks (for early diagnosis of destructive thyroiditis or persistent thyrotoxicosis)
	• without or with mild symptoms	
	• TSH < 0.4 mlU/l	Stop ICIs if symptoms are present until they subside; Consider b blockers and methimazole
		If thyrotoxicosis persists (>6 weeks), investigate for Grave’s searching TRab, testing fT3, conducting thyroid scintigraphy or ultrasound; hydrate and provide supportive care
	• moderate symptoms	
	• TSH < 0.4 mlU/l	Manage similarly as mentioned right above; Corticosteroids may be used
	• severe symptoms	
	• TSH < 0.4 mlU/l	Manage similarly as mentioned right above; Hospitalization may be needed for thyroid storm concern
	• very severe symptoms (life threatening*)*	
Thyrotoxic crisis		Hold ICIs; Give non-selective b blockers, high-dose glucocorticoids and PTU; monitor TSH and FT4 levels 4–6 weeks after the crisis; If normal, the management is completed. If not, search for TRAb, even test fT3, administer I-123 or conduct a scan to identify the cause of persistent (>6 weeks) thyrotoxicosis and exclude Graves’ disease
Thyroid-Like Eye Disease (TED)		Hold ICIs only in severe forms of TED; Give systemic corticosteroids in high dosages; Canthotomy/cantholysis for severe cases

*PTU* propylthiouracil, *fT4* free thyroxine, *TSH* thyroid-stimulating hormone, *ICI* immune checkpoint inhibitor, *TRab* TSH Receptor antibody, *fT3* free triiodothyronine

## Conclusion

The ICI therapy has revolutionized cancer treatment by harnessing the immune system to fight malignancies. While offering promising therapeutic results and improved survival rates for cancer patients, the utilization of ICIs is associated with various adverse effects, among which thyroid disorders are notably prevalent. The pathogenesis of ICI-induced thyroid dysfunction encompasses immune-mediated acute inflammation leading to destructive thyroiditis. T cell activation, alongside the involvement of various antibodies and cytokines, plays a significant role in both initiating and progressing the disease [1, 8, 16, 17].

Diagnosing ICI-related thyroid disorders requires vigilance and regular thyroid function screening, utilizing measurements of TSH, fT4, and TRAb, TPO-antibodies and TSH receptor antibodies, according to specific guidelines [13, 14, 53–57]. Clinical presentations vary from asymptomatic cases to severe manifestations of thyrotoxicosis, hypothyroidism, and even Graves’ disease [24, 30, 32]. Early detection demands a thorough examination encompassing both clinical and laboratory evaluation.

Understanding the pathophysiological mechanisms underlying these adverse effects is vital for the development of effective treatment strategies. The management of these thyroid-related side effects necessitates an individualized approach that considers the severity of the condition, the patient’s clinical state, and the stage of any malignancy involved. As such, a collaborative effort between various medical professionals is essential to ensure optimal care [13, 14, 53, 54, 56]. Guidelines established by prominent organizations such as ASCO, NCCN, SITC, and ESMO serve as a valuable resource for healthcare providers, highlighting the significance of tailored treatments based on the severity grade.

Close monitoring, prompt diagnosis, and personalized treatment strategies are crucial for addressing the complexities of ICI-induced thyroid disorders. Collaboration between healthcare professionals and continuous research are essential for the formulation of future guidelines, the implementation of tailored treatment and the enhancement of ICI therapy.

## References

[1] Husebye ES, Castinetti F, Criseno S, Curigliano G, Decallonne B, Fleseriu M, Higham CE, Lupi I, Paschou SA, Toth M, van der Kooij M, Dekkers OM (2022). Endocrine-related adverse conditions in patients receiving immune checkpoint inhibition: An ESE clinical practice guideline. Eur. J. Endocrinol..

[2] Moslehi J, Lichtman AH, Sharpe AH, Galluzzi L, Kitsis RN (2021). Immune checkpoint inhibitor-associated myocarditis: Manifestations and mechanisms. J. Clin. Investig..

[3] Okura N, Asano M, Uchino J, Morimoto Y, Iwasaku M, Kaneko Y, Yamada T, Fukui M, Takayama K (2020). Endocrinopathies associated with immune checkpoint inhibitor cancer treatment: A review. J. Clin. Med..

[4] Z. Cardona, J.A. Sosman, S. Chandra, W Huang, Endocrine side effects of immune checkpoint inhibitors. Front Endocrinol 14 (2023). https://www.frontiersin.org/articles/10.3389/fendo.2023.1157805 10.3389/fendo.2023.1157805 PMC10210589 37251665

[5] Morganstein DL, Lai Z, Spain L, Diem S, Levine D, Mace C, Gore M, Larkin J (2017). Thyroid abnormalities following the use of cytotoxic T-lymphocyte antigen-4 and programmed death receptor protein-1 inhibitors in the treatment of melanoma. Clin. Endocrinol..

[6] Lee H, Hodi FS, Giobbie-Hurder A, Ott PA, Buchbinder EI, Haq R, Tolaney S, Barroso-Sousa R, Zhang K, Donahue H, Davis M, Gargano ME, Kelley KM, Carroll RS, Kaiser UB, Min L (2017). Characterization of thyroid disorders in patients receiving immune checkpoint inhibition therapy. Cancer Immunol. Res..

[7] Guaraldi F, La Selva R, Samà MT, D’Angelo V, Gori D, Fava P, Fierro MT, Savoia P, Arvat E (2018). Characterization and implications of thyroid dysfunction induced by immune checkpoint inhibitors in real-life clinical practice: A long-term prospective study from a referral institution. J. Endocrinol. Investig..

[8] Kotwal A, Gustafson MP, Bornschlegl S, Kottschade L, Delivanis DA, Dietz AB, Gandhi M, Ryder M (2020). Immune checkpoint inhibitor-induced thyroiditis is associated with increased intrathyroidal T lymphocyte subpopulations. Thyroid.

[9] de Filette J, Jansen Y, Schreuer M, Everaert H, Velkeniers B, Neyns B, Bravenboer B (2016). Incidence of thyroid-related adverse events in melanoma patients treated with pembrolizumab. J. Clin. Endocrinol. Metab..

[10] Azmat U, Liebner D, Joehlin-Price A, Agrawal A, Nabhan F (2016). Treatment of ipilimumab induced graves’ disease in a patient with metastatic melanoma. Case Rep. Endocrinol..

[11] Gan EH, Mitchell AL, Plummer R, Pearce S, Perros P (2017). Tremelimumab-induced graves hyperthyroidism. Eur. Thyroid J..

[12] Deligiorgi MV, Sagredou S, Vakkas L, Trafalis DT (2021). The continuum of thyroid disorders related to immune checkpoint inhibitors: STill Many Pending Queries. Cancers.

[13] Paschou SA, Stefanaki K, Psaltopoulou T, Liontos M, Koutsoukos K, Zagouri F, Lambrinoudaki I, Dimopoulos M-A (2021). How we treat endocrine complications of immune checkpoint inhibitors. ESMO Open.

[14] Thompson JA, Schneider BJ, Brahmer J, Andrews S, Armand P, Bhatia S, Budde LE, Costa L, Davies M, Dunnington D, Ernstoff MS, Frigault M, Hoffner B, Hoimes CJ, Lacouture M, Locke F, Lunning M, Mohindra NA, Naidoo J, Scavone JL (2019). Management of immunotherapy-related toxicities, Version 1.2019, NCCN clinical practice guidelines in oncology. J. Natl. Compr. Cancer Netw..

[15] Yuen KCJ, Samson SL, Bancos I, Gosmanov AR, Jasim S, Fecher LA, Weber JS (2022). American association of clinical endocrinology disease state clinical review: Evaluation and management of immune checkpoint inhibitor-mediated endocrinopathies: A practical case-based clinical approach. Endocr. Pract..

[16] Torimoto K, Okada Y, Nakayamada S, Kubo S, Tanaka Y (2017). Anti-PD-1 antibody therapy induces hashimoto’s disease with an increase in peripheral blood follicular helper T cells. Thyroid.

[17] Maekura T, Naito M, Tahara M, Ikegami N, Kimura Y, Sonobe S, Kobayashi T, Tsuji T, Minomo S, Tamiya A, Atagi S (2017). Predictive factors of nivolumab-induced hypothyroidism in patients with non-small cell lung cancer. Vivo.

[18] Osorio JC, Ni A, Chaft JE, Pollina R, Kasler MK, Stephens D, Rodriguez C, Cambridge L, Rizvi H, Wolchok JD, Merghoub T, Rudin CM, Fish S, Hellmann MD (2017). Antibody-mediated thyroid dysfunction during T-cell checkpoint blockade in patients with non-small-cell lung cancer. Ann. Oncol..

[19] Muir CA, Menzies AM, Clifton-Bligh R, Tsang VHM (2020). Thyroid toxicity following immune checkpoint inhibitor treatment in advanced cancer. Thyroid.

[20] Zhou X, Iwama S, Kobayashi T, Ando M, Arima H (2023). Risk of thyroid dysfunction in PD-1 blockade is stratified by the pattern of TgAb and TPOAb positivity at baseline. J. Clin. Endocrinol. Metab..

[21] Delivanis DA, Gustafson MP, Bornschlegl S, Merten MM, Kottschade L, Withers S, Dietz AB, Ryder M (2017). Pembrolizumab-induced thyroiditis: Comprehensive clinical review and insights into underlying involved mechanisms. J. Clin. Endocrinol. Metab..

[22] Kurimoto C, Inaba H, Ariyasu H, Iwakura H, Ueda Y, Uraki S, Takeshima K, Furukawa Y, Morita S, Yamamoto Y, Yamashita S, Katsuda M, Hayata A, Akamatsu H, Jinnin M, Hara I, Yamaue H, Akamizu T (2020). Predictive and sensitive biomarkers for thyroid dysfunctions during treatment with immune-checkpoint inhibitors. Cancer Sci..

[23] L. Zhan, H. Feng, H. Liu, L. Guo, C. Chen, X. Yao, S Sun, Immune checkpoint inhibitors-related thyroid dysfunction: Epidemiology, clinical presentation, possible pathogenesis, and management. Front. Endocrinol. 12 (2021). https://www.frontiersin.org/articles/10.3389/fendo.2021.649863 10.3389/fendo.2021.649863 PMC8224170 34177799

[24] Brancatella A, Viola N, Brogioni S, Montanelli L, Sardella C, Vitti P, Marcocci C, Lupi I, Latrofa F (2019). Graves’ disease induced by immune checkpoint inhibitors: A case report and review of the literature. Eur. Thyroid J..

[25] Olsson-Brown A, Lord R, Sacco J, Wagg J, Coles M, Pirmohamed M (2020). Two distinct clinical patterns of checkpoint inhibitor-induced thyroid dysfunction. Endocr. Connect..

[26] Lu D, Yao J, Yuan G, Gao Y, Zhang J, Guo X (2022). Immune checkpoint inhibitor-related new-onset thyroid dysfunction: A retrospective analysis using the US FDA adverse event reporting system. Oncologist.

[27] Stelmachowska-Banaś M, Czajka-Oraniec I (2020). Management of endocrine immune-related adverse events of immune checkpoint inhibitors: An updated review. Endocr. Connect..

[28] Goyal I, Pandey MR, Sharma R, Chaudhuri A, Dandona P (2021). The side effects of immune checkpoint inhibitor therapy on the endocrine system. Indian J. Med. Res..

[29] Del Rivero J, Cordes LM, Klubo‐Gwiezdzinska J, Madan RA, Nieman LK, Gulley JL (2020). Endocrine‐related adverse events related to immune checkpoint inhibitors: Proposed algorithms for management. Oncologist.

[30] Chang L-S, Barroso-Sousa R, Tolaney SM, Hodi FS, Kaiser UB, Min L (2019). Endocrine toxicity of cancer immunotherapy targeting immune checkpoints. Endocr. Rev..

[31] J. Villadolid, A. Amin, Immune checkpoint inhibitors in clinical practice: Update on management of immune-related toxicities. Transl. Lung Cancer Res. 4(5) (2015). 10.3978/j.issn.2218-6751.2015.06.06 10.3978/j.issn.2218-6751.2015.06.06 PMC4630514 26629425

[32] Duan L, Wang L, Wang H, Si X, Zhang L, Liu X, Li Y, Guo X, Zhou J, Zhu H, Zhang L (2020). Clinical diagnosis and treatment of immune checkpoint inhibitors-related endocrine dysfunction. Thorac. Cancer.

[33] S. A. Paschou, M. Liontos, E. Eleftherakis-Papaiakovou, K. Stefanaki, C. Markellos, K. Koutsoukos, F. Zagouri, T. Psaltopoulou, M.-A Dimopoulos, Oncological patients with endocrine complications after immunotherapy with checkpoint inhibitors present longer progression-free and overall survival. Front. Oncol. 12 (2022). https://www.frontiersin.org/articles/10.3389/fonc.2022.847917 10.3389/fonc.2022.847917 PMC8987508 35402216

[34] Anderson B, Morganstein DL (2021). Endocrine toxicity of cancer immunotherapy: Clinical challenges. Endocr. Connect..

[35] Wright JJ, Powers AC, Johnson DB (2021). Endocrine toxicities of immune checkpoint inhibitors. Nat. Rev. Endocrinol..

[36] Kassi E, Angelousi A, Asonitis N, Diamantopoulos P, Anastasopoulou A, Papaxoinis G, Kokkinos M, Giovanopoulos I, Kyriakakis G, Petychaki F, Savelli A, Benopoulou O, Gogas H (2019). Endocrine-related adverse events associated with immune-checkpoint inhibitors in patients with melanoma. Cancer Med..

[37] Chera A, Stancu AL, Bucur O (2022). Thyroid-related adverse events induced by immune checkpoint inhibitors. Front. Endocrinol..

[38] Yang S, Yu K-H, Palmer N, Fox K, Kou SC, Kohane IS (2020). Autoimmune effects of lung cancer immunotherapy revealed by data-driven analysis on a nationwide cohort. Clin. Pharmacol. Ther..

[39] Migden MR, Rischin D, Schmults CD, Guminski A, Hauschild A, Lewis KD, Chung CH, Hernandez-Aya L, Lim AM, Chang ALS, Rabinowits G, Thai AA, Dunn LA, Hughes BGM, Khushalani NI, Modi B, Schadendorf D, Gao B, Seebach F, Fury MG (2018). PD-1 blockade with cemiplimab in advanced cutaneous squamous-cell carcinoma. N. Engl. J. Med..

[40] L. Trudu, G. Guaitoli, F. Bertolini, M. Maur, C. Santini, V. R. Papapietro, S. Talerico, S. Natalizio, C. Isca, M. Dominici, F. Barbieri, Thyroid function impairment after chemo-immunotherapy for advanced NSCLC: A single institutional retrospective report. Immunotherapy (2022). 10.2217/imt-2021-0165 10.2217/imt-2021-0165 35416048

[41] Prather LL, Ali A, Wang H, Du W, Chen N, Sheth A, Sandulache V, Sabichi AL, Kemnade JO, Wang DY (2022). Immune-related adverse events and immunotherapy efficacy in patients with cancer: A retrospective study. J. Clin. Oncol..

[42] Zheng X, Xiao H, Long J, Wei Q, Liu L, Zan L, Ren W (2022). Dynamic follow-up of the effects of programmed death 1 inhibitor treatment on thyroid function and sonographic features in patients with hepatocellular carcinoma. Endocr. Connect..

[43] Baek H-S, Jeong C, Shin K, Lee J, Suh H, Lim D-J, Kang MI, Ha J (2022). Association between the type of thyroid dysfunction induced by immune checkpoint inhibitors and prognosis in cancer patients. BMC Endocr. Disord..

[44] Kotwal A, Kottschade L, Ryder M (2020). PD-L1 inhibitor-induced thyroiditis is associated with better overall survival in cancer patients. Thyroid.

[45] Lima Ferreira J, Costa C, Marques B, Castro S, Victor M, Oliveira J, Santos AP, Sampaio IL, Duarte H, Marques AP, Torres I (2021). Improved survival in patients with thyroid function test abnormalities secondary to immune-checkpoint inhibitors. Cancer Immunol. Immunotherapy.

[46] Li X, Wang X, Wang S, Liu Y, Wang R, Liu Y, Huang L, Feng Y, Xie X, Shi L (2023). Thyroid dysfunction induced by immune checkpoint inhibitors and tumor progression during neoadjuvant therapy of non‑small cell lung cancer: A case report and literature review. Oncol. Lett..

[47] Petranović Ovčariček P, Verburg FA, Hoffmann M, Iakovou I, Mihailovic J, Vrachimis A, Luster M, Giovanella L (2021). Correction to: Higher thyroid hormone levels and cancer. Eur. J. Nucl. Med. Mol. Imaging.

[48] von Itzstein MS, Gonugunta AS, Wang Y, Sheffield T, Lu R, Ali S, Fattah FJ, Xie D, Cai J, Xie Y, Gerber DE (2022). Correction to: Divergent prognostic effects of pre-existing and treatment-emergent thyroid dysfunction in patients treated with immune checkpoint inhibitors. Cancer Immunol., Immunotherapy: CII.

[49] Coniac S, Stoian M (2021). Updates in endocrine immune-related adverse events in oncology immunotherapy. Acta Endocrinologica (Buchar., Rom.: 2005).

[50] Byun DJ, Wolchok JD, Rosenberg LM, Girotra M (2017). Cancer immunotherapy—Immune checkpoint blockade and associated endocrinopathies. Nat. Rev. Endocrinol..

[51] Hamnvik O-PR, Larsen PR, Marqusee E (2011). Thyroid dysfunction from antineoplastic agents. J. Natl. Cancer Inst..

[52] Girotra M, Hansen A, Farooki A, Byun DJ, Min L, Creelan BC, Callahan MK, Atkins MB, Sharon E, Antonia SJ, West P, Gravell AE, Investigational Drug Steering Committee (IDSC) Immunotherapy Task Force collaboration (2018). The current understanding of the endocrine effects from immune checkpoint inhibitors and recommendations for management. JNCI Cancer Spectr..

[53] Brahmer JR, Lacchetti C, Schneider BJ, Atkins MB, Brassil KJ, Caterino JM, Chau I, Ernstoff MS, Gardner JM, Ginex P, Hallmeyer S, Holter Chakrabarty J, Leighl NB, Mammen JS, McDermott DF, Naing A, Nastoupil LJ, Phillips T, Porter LD, National Comprehensive Cancer Network (2018). Management of immune-related adverse events in patients treated with immune checkpoint inhibitor therapy: American society of clinical oncology clinical practice guideline. J. Clin. Oncol.

[54] Puzanov I, Diab A, Abdallah K, Bingham CO, Brogdon C, Dadu R, Hamad L, Kim S, Lacouture ME, LeBoeuf NR, Lenihan D, Onofrei C, Shannon V, Sharma R, Silk AW, Skondra D, Suarez-Almazor ME, Wang Y, Wiley K, Zheng P (2017). Managing toxicities associated with immune checkpoint inhibitors: Consensus recommendations from the Society for Immunotherapy of Cancer (SITC) Toxicity Management Working Group. J. Immunother. Cancer.

[55] Arima H, Iwama S, Inaba H, Ariyasu H, Makita N, Otsuki M, Kageyama K, Imagawa A, Akamizu T (2019). Management of immune-related adverse events in endocrine organs induced by immune checkpoint inhibitors: Clinical guidelines of the Japan Endocrine Society. Endocr. J..

[56] Castinetti F, Albarel F, Archambeaud F, Bertherat J, Bouillet B, Buffier P, Briet C, Cariou B, Caron P, Chabre O, Chanson P, Cortet C, Cao CD, Drui D, Haissaguerre M, Hescot S, Illouz F, Kuhn E, Lahlou N, Borson-Chazot F (2019). French Endocrine Society Guidance on endocrine side effects of immunotherapy. Endocr.-Relat. Cancer.

[57] Higham CE, Olsson-Brown A, Carroll P, Cooksley T, Larkin J, Lorigan P, Morganstein D, Trainer PJ (2018). Society for endocrinology endocrine emergency guidance: Acute management of the endocrine complications of checkpoint inhibitor therapy. Endocr. Connect..

[58] Yu CW, Yau M, Mezey N, Joarder I, Micieli JA (2020). Neuro-ophthalmic complications of immune checkpoint inhibitors: A systematic review. Eye Brain.

